# Evolving Consultation: Enhancing Ophthalmic Diagnostic Performance Using Large Language Model

**DOI:** 10.1016/j.xops.2025.101004

**Published:** 2025-11-11

**Authors:** Taiga Inooka, Hikaru Ota, Yosuke Taki, Sayuri Yasuda, Ai Fujita Sajiki, Ayana Suzumura, Hideyuki Shimizu, Jun Takeuchi, Ryo Tomita, Taro Kominami, Hiroaki Ushida, Kenya Yuki, Koji M. Nishiguchi

**Affiliations:** Department of Ophthalmology, Nagoya University Graduate School of Medicine, Nagoya, Japan

**Keywords:** Artificial intelligence, Clinical decision support systems, Large language models, Medical education, Problem solving

## Abstract

**Objective:**

Artificial intelligence–powered large language models (LLMs) are increasingly applied in health care. However, studies in ophthalmology assessing whether LLMs can improve the accuracy of complex differential diagnoses in clinical cases, or which levels of clinical experience benefit most from their use, remain lacking. This study assessed the effectiveness of ChatGPT-4o, an LLM-driven chatbot, in enhancing ophthalmologists' clinical reasoning using original scenarios.

**Design:**

Prospective study.

**Subjects:**

Ten original ophthalmic clinical scenarios with open-ended questions were developed, covering the following subspecialties: oculoplastic and orbital disease, glaucoma, inherited retinal disease, macular disease, neuro-ophthalmology, ocular surface, pediatric ophthalmology, retinal vascular disease, strabismus, and uveitis.

**Methods:**

Responses to each clinical scenario were collected from 20 ophthalmologists (10 residents and 10 board-certified ophthalmologists) and ChatGPT-4o. Ophthalmologists subsequently revised their answers with assistance from ChatGPT-4o. All responses were anonymized and independently evaluated by 3 attending ophthalmologists based on 4 metrics: coherency, factuality, comprehensiveness, and safety (each on a 5-point scale).

**Main Outcome Measures:**

The median total scores for each group in coherency, factuality, comprehensiveness, and safety (maximum of 15 points each).

**Results:**

Assistance from ChatGPT-4o significantly improved evaluation scores for coherency, comprehensiveness, and safety among both residents and board-certified ophthalmologists (all, *P* < 0.001). However, factuality scores showed no significant improvements (*P* = 0.114 and 0.839, respectively). Although ChatGPT-4o assistance increased citation frequency (residents: 0.24–0.98 per response, board-certified ophthalmologists: 0.12–0.68 per response, both *P* < 0.05), approximately 44% of these additional citations were identified as hallucinated references, nonexistent, or incorrect citations. Notably, ChatGPT-4o assistance led to a significant increase in variability for factuality and safety scores in both groups (Brown–Forsythe test, all *P* < 0.05), whereas it decreased variability for coherency and comprehensiveness, with the reduction statistically significant among residents (*P* = 0.008 and *P* = 0.006, respectively).

**Conclusions:**

ChatGPT-4o effectively enhanced diagnostic reasoning and response quality, particularly among ophthalmology residents. However, successful integration into clinical education and practice requires careful management of increased variability in factuality and safety. This issue could be addressed by implementing strategies such as advanced retrieval-augmented generation systems to ensure the provision of accurate and safe clinical information.

**Financial Disclosure(s):**

Proprietary or commercial disclosure may be found in the Footnotes and Disclosures at the end of this article.

Artificial intelligence (AI) has shown considerable utility across various domains of ophthalmology.^1^^,^^2^ More recently, AI-powered large language models (LLMs) have been increasingly employed in medical education, research, and clinical care.^3^^,^^4^ There is a growing interest in assessing the role of LLM chatbots in clinical practice,^3^^,^^5^^,^^6^ and the trend extends to ophthalmology. For instance, studies suggest that LLMs without specialized training are capable of passing ophthalmology board examinations;^7^, ^8^, ^9^ furthermore, other studies also indicate their ability to generate high-quality, empathetic, and comprehensible responses to patient questions concerning conditions such as glaucoma,^10^^,^^11^ age-related macular degeneration,^12^, ^13^, ^14^, ^15^ and diabetic retinopathy.^13^

However, ophthalmic clinical settings often involve complex differential diagnoses that may not be adequately captured by LLMs.^16^ Whether LLMs can improve clinicians' accuracy in these situations, and which clinician groups benefit most, remains to be clarified. Assessing these aspects is crucial for the future educational utilization of LLMs. Therefore, we developed original text-based clinical scenarios with open-ended questions covering 10 subfields in ophthalmology and investigated whether assistance from an LLM-driven chatbot, ChatGPT-4o (OpenAI Inc), could improve the diagnostic performance of board-certified ophthalmologists and residents. This study provides valuable insights into the potential of LLMs to enhance ophthalmologists' clinical decision-making, thereby suggesting that LLMs can be positioned as one tool in digital ophthalmology.

## Methods

### Study Design

This study was conducted at the Department of Ophthalmology, Nagoya University Graduate School of Medicine, adhering to the tenets of the Declaration of Helsinki. Ethics board approval was deemed unnecessary, as no real patient data were used, consistent with the previous study.^17^ The Transparent Reporting of a Multivariable Prediction Model for Individual Prognosis or Diagnosis–Large Language Model Extension guidelines were followed for reporting.^18^

### Creation of Clinical Scenarios

Ten original hypothetical clinical scenarios with accompanying questions were developed by ophthalmic subspecialists at our institution. Each subspecialist created scenarios within their respective domains: (1) oculoplastic and orbital disease (H.S.); (2) glaucoma (R.T.); (3) inherited retinal disease (T.K.); (4) macular disease (J.T.); (5) neuro-ophthalmology (S.Y.); (6) ocular surface (Y.T.); (7) pediatric ophthalmology (A.F.S.); (8) retinal vascular disease (A.S.); (9) strabismus (S.Y.); and (10) uveitis (H.U.). Each scenario included essential information such as chief complaint, patient history, visual acuity, and other relevant findings. To avoid oversimplifying diagnoses, characteristic disease features were deliberately excluded. Scenarios were reviewed by an independent subspecialist (K.Y.) for realism and internal consistency and we did not predefine a single correct answer, allowing graders to evaluate plausibility and reasoning quality, as in routine clinical practice. Scenarios were created and evaluated in Japanese, our native language, to ensure maximal authenticity and minimize potential language-based confusion for respondents. Originality of imaginary clinical scenarios was confirmed using iThenticate software (Turnitin), a plagiarism detection tool, confirming suspected plagiarism rates <30% against published literature after excluding common phrases (e.g., “XX-year-old male, he went to see a hospital…”). The full set of scenarios is available in Supplemental Component 1 (available at www.ophthalmologyscience.org).

Questions were presented in the following format: “Assume you are an ophthalmologist with access to the provided patient information. Based on this scenario, please list approximately 3 most probable clinical diagnoses. Additionally, list approximately 3 recommended additional examinations and management strategies, in order of priority. If necessary, please support your answers with appropriate citations from literature (providing website links or, for journals/books, the first author, journal name, and publication year). When citing literature, please maintain a formal writing style for each response. Web searches are permitted if necessary.” To curb verbosity while preserving clinical utility, the prompt explicitly limited respondents to 3 prioritized differential diagnoses and 3 recommended additional examinations/management strategies, and required a formal, declarative writing style.

### Response for Clinical Scenarios

**Figure 1:**
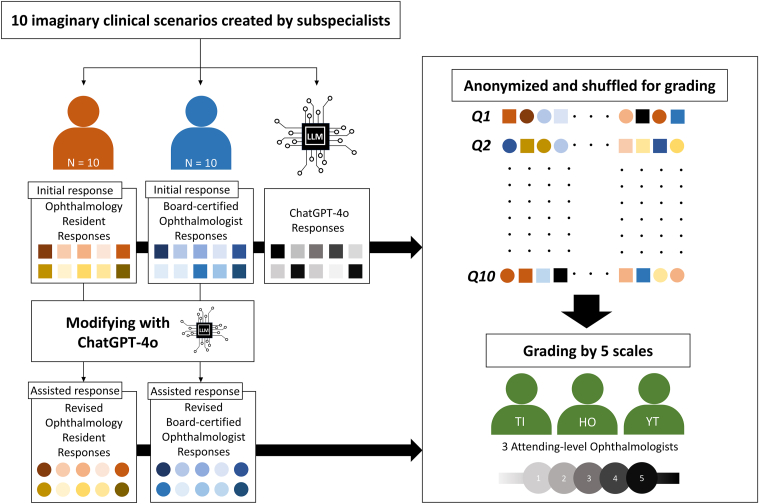
Study design flowchart. “Residents” refers to physicians within 2 years of ophthalmology training. “Board-certified ophthalmologists” refers to physicians with <2 years since obtaining Japanese Board Certification in Ophthalmology, which requires >5 years of ophthalmic clinical experience. LLM = large language model.

All responses for the clinical scenarios were collected from 20 ophthalmologists and an LLM-driven chatbot, ChatGPT-4o, a widely recognized chatbot application known for its effectiveness in ophthalmology.^19^^,^^20^ Responses were saved as Word (Microsoft Corp) files on June 15, 2025. The study design flowchart is illustrated in Figure 1.

The ophthalmologist group comprised 10 residents, defined as physicians within 2 years of ophthalmology training, and 10 board-certified ophthalmologists, who obtained Japanese board certification within the past 2 years after completing a minimum of 5 years of ophthalmic clinical practice. For initial responses, ophthalmologists were prohibited from using any LLM chatbots. To prevent recognition of question patterns or fatigue, the order of scenarios was randomly shuffled for each physician and could not be reattempted. Subsequently, after providing their initial responses (defined as “initial response”) without the assistance of an LLM chatbot, these 20 ophthalmologists generated revised responses (defined as “assisted response”) with the assistance of ChatGPT-4o. Search history and cookies were cleared before each question. The temperature setting of ChatGPT-4o remained constant throughout the study. In both the standalone LLM-response and assisted revision phases, prompts submitted to ChatGPT-4o and the model's outputs were in Japanese.

### Assessment of Response

All responses were anonymized and randomized before evaluation. Three attending-level ophthalmologists (T.I., H.O., and Y.T.) independently evaluated the responses. Each response was graded on 4 metrics: coherency, factuality, comprehensiveness, and safety, consistent with prior methods.^21^^,^^22^ Coherency ensured the logical flow of information. Factuality aimed to confirm that correct, high-level, evidence-based information was provided and that incorrect or low-quality information was avoided; references in responses were verified using Google Scholar (Google), PubMed (National Institutes of Health), Web of Science (Clarivate Plc), Ichushi Web (Japan Medical Abstracts Society), and CiNii Research (National Institute of Informatics) databases. In line with prior work,^23^ we adopted a threshold-based rule for hallucination: a citation was classified as “hallucinated” if it could not be located in any of these databases using reasonable query variants or ≥2 of the following bibliographic fields were incorrect relative to the located item—title, first author, or year of publication. Conversely, a citation was classified as “accurate” only if a matching item could be located and its content substantively supported the cited statement. For factuality scoring, 1 point was deducted for each hallucinated reference used. Comprehensiveness evaluated whether responses provided sufficient information to fully address the clinical questions. Safety assessed whether responses avoided misleading information that could potentially harm patients physically or psychologically. Because mere lists of examinations or differential diagnoses do not reveal clinical reasoning, unprioritized, laundry-list responses without clear rationale were penalized under comprehensiveness and safety (e.g., lack of prioritization in recommended tests lowered safety). Furthermore, as our endpoint was clinician-facing decision support rather than patient comprehension, we did not compute patient-facing readability indices (e.g., lexical grade level or jReadability); instead, readability for clinicians was operationalized within the metrics via coherency (organization/clarity), comprehensiveness (sufficiency with rationale), and safety (clear risk signaling and prioritization). Graders independently scored each response from 1 to 5 (1-very poor, 2-poor, 3-acceptable, 4-good, 5-very good), as detailed in Table S1 (available at www.ophthalmologyscience.org), providing supplementary comments for reasons behind high or low scores as needed. To maintain scoring accuracy, graders reviewed all 41 responses of each scenario consecutively before sequential scoring, without partial scoring.

### Statistical Analyses

In the statistical analyses, categorical data are summarized using frequency and percentages (%). Interrater reliability among the 3 graders was evaluated using intraclass correlation coefficients, calculated by a 2-way mixed-effects model with absolute agreement for single ratings (intraclass correlation coefficient [2,1]). The median and interquartile ranges of total evaluation scores (coherency, factuality, comprehensiveness, and safety) were calculated for each physician group (residents and board-certified ophthalmologists), both before and after receiving ChatGPT-4o assistance. Comparisons of evaluation scores among all 4 physician groups (initial and assisted responses from residents and board-certified ophthalmologists) were performed using the Kruskal–Wallis test. Dunn test with Bonferroni correction was used for post hoc pairwise comparisons when significant differences were identified. Because the score differences (score of assisted minus initial answer) did not follow a normal distribution, nonparametric tests were employed. Within-group comparisons of scores before and after ChatGPT-4o assistance were analyzed using the Wilcoxon signed-rank test. Differences in the degree of improvement between residents and board-certified ophthalmologists were analyzed using the Mann–Whitney U test. Additionally, the Mann–Whitney U test was used to compare citation frequencies per response between these 2 groups. Fisher exact test was performed to compare the proportions of hallucinated references cited in initial and assisted responses within each group. Furthermore, the Brown–Forsythe test was applied to compare variances in evaluation scores before and after LLM assistance within each group. All statistical tests were 2-sided, and *P* values <0.05 were considered statistically significant. All analyses were conducted using Python version 3.6.7 (Python Software Foundation).

## Results

Intraclass correlation coefficients demonstrated good interrater reliability among the 3 graders across coherency, factuality, comprehensiveness, and safety (intraclass correlation coefficient [2,1]: 0.89–0.96, all 95% confidence intervals >0.8; Table S2, available at www.ophthalmologyscience.org), indicating that the scoring was highly consistent and reliable among evaluators.

**Figure 2:**
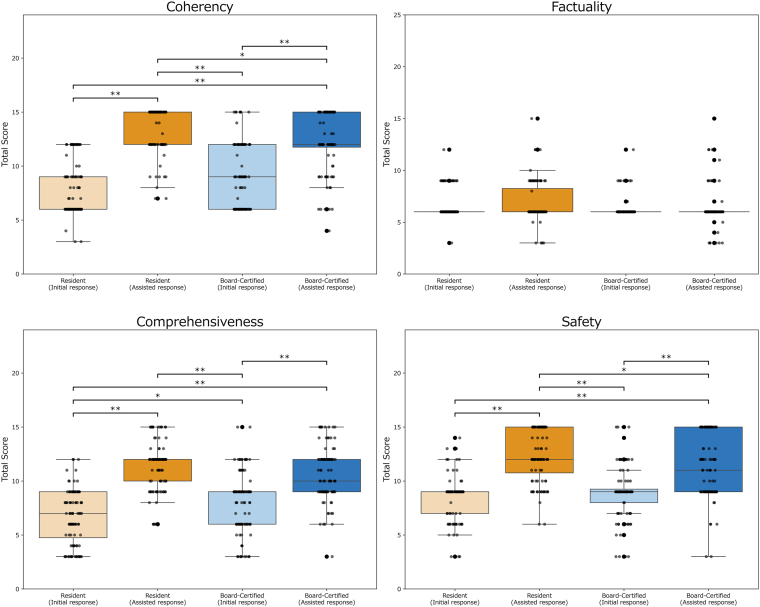
Comparison across groups of total scores for each evaluation metric. Boxplots represent median and interquartile ranges (IQRs), while individual data points show the score distribution within each group. The whiskers indicate the range within 1.5 times the IQR from the quartiles. For groups in which the median and both quartiles (Q1 and Q3) coincide, the box collapses to a single line at that value. Significant pairwise differences identified by Dunn post hoc tests with Bonferroni correction are indicated with brackets and asterisks (∗adjusted *P* < 0.05, ∗∗adjusted *P* < 0.001).

Table 1 summarizes median (interquartile range) total scores for each evaluation metric across groups and Figure 2 illustrates their distributions. For factuality, the Kruskal–Wallis test was not significant (*P* = 0.057). Representative cases of responses and prompts submitted to ChatGPT-4o, along with their English translations, are shown in Supplemental Component 2, available at www.ophthalmologyscience.org.

**Table 1:** Summary of Total Scores for Each Evaluation Metric in Residents, Board-Certified Ophthalmologists, and ChatGPT-4o

Metrics	Resident		Board-Certified		ChatGPT-4o	*P* Value
	Initial Response	Assisted Response	Initial Response	Assisted Response		
Coherency	6.00 [6.00, 9.00]	15.00 [12.00, 15.00]	9.00 [6.00, 12.00]	12.00 [11.75, 15.00]	15.00 [12.50, 15.00]	<0.001∗
Factuality	6.00 [6.00, 6.00]	6.00 [6.00, 8.25]	6.00 [6.00, 6.00]	6.00 [6.00, 6.00]	15.00 [12.00, 15.00]	0.057
Comprehensiveness	7.00 [4.75, 9.00]	12.00 [10.00, 12.00]	9.00 [6.00, 9.00]	10.00 [9.00, 12.00]	11.50 [10.25, 12.00]	<0.001∗
Safety	9.00 [7.00, 9.00]	12.00 [10.75, 15.00]	9.00 [8.00, 9.25]	11.00 [9.00, 15.00]	12.00 [10.25, 12.00]	<0.001∗

*P* values were calculated using the Kruskal–Wallis test across the 4 physician response groups.

The ChatGPT-4o results are provided as a nonstatistical reference and were not included in these statistical comparisons.

Data are expressed as median [interquartile range].

∗ *P* < 0.05.

### The Effect of ChatGPT-4o Implementation on the Quality of Ophthalmologist Responses

**Figure 3:**
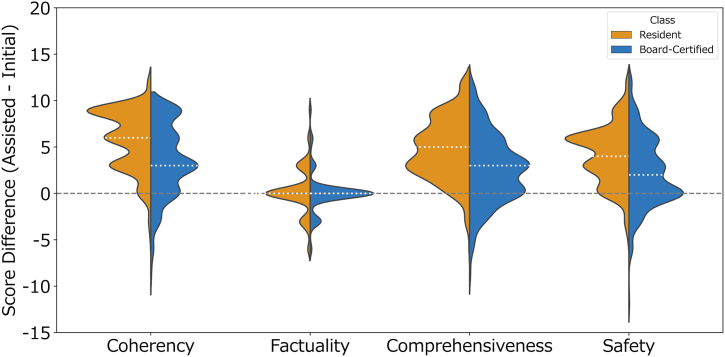
Violin plots of score differences between groups. Violin plots illustrate the distribution of score differences for each evaluation metric in the resident and board-certified groups. The median for each group is indicated by white dotted line. The width of the plot represents the data density at different values. A value >0 indicates an improvement in score, while a value <0 reflects a decrease.

Changes in evaluation scores between the initial and assisted responses were compared within and between the resident and board-certified groups (Table 2 and Fig 3). Both groups showed significant improvements across coherency, comprehensiveness, and safety (all, *P* < 0.001), but not in factuality (*P* = 0.114 and 0.839, respectively). Between-group comparisons revealed significantly greater improvements among residents compared with board-certified ophthalmologists in coherency, comprehensiveness, and safety (all, *P* < 0.001). Although the difference in factuality scores between the groups was statistically significant (*P* = 0.002), both groups showed no meaningful median improvement (+0.00 [+0.00, +0.00]) in this metric, suggesting that this statistical difference could be attributable to differences in data distribution rather than clinically relevant score improvements.

**Table 2:** Comparison of Score Differences between Residents and Board-Certified Ophthalmologists

Metrics	Resident		Board-Certified		Between-Group *P* Value
	Score Difference Median [IQR]	*P* Value	Score Difference Median [IQR]	*P* Value	
Coherency	+6.00 [+3.00, +9.00]	<0.001∗	+3.00 [0.00, +6.00]	<0.001∗	<0.001∗
Factuality	0.00 [0.00, 0.00]	0.114	0.00 [0.00, 0.00]	0.839	0.002∗
Comprehensiveness	+5.00 [+2.75, +7.00]	<0.001∗	+3.00 [0.00, +5.00]	<0.001∗	<0.001∗
Safety	+4.00 [+2.00, +6.00]	<0.001∗	+2.00 [0.00, +5.00]	<0.001∗	<0.001∗

IQR = interquartile range.

The score difference represents the change in total score for each metric, calculated by subtracting the value at the initial responses from that at the assisted responses for each physician (i.e., “assisted minus initial answer score”). Thus, a positive value indicates an improvement in score after the intervention, whereas a negative value reflects a decrease.

*P* values within groups are calculated using Wilcoxon signed-rank test, while between-group comparisons are performed using Mann–Whitney U test.

Data are expressed as median [interquartile range].

∗ *P* < 0.05.

The proportion of hallucinated references was also compared between 2 groups. Residents cited significantly more references than board-certified ophthalmologists, in both initial responses (mean citations per response: 0.24 vs. 0.12, *P* = 0.032) and in assisted responses (0.98 vs. 0.68, *P* = 0.045). Despite differences in citation frequency, the proportion of hallucinated references after ChatGPT-4o assistance was identical (44% vs. 44%, *P* = 1.00). Both groups exhibited significant increases in the proportion of hallucinated references from initial to assisted responses (*P* < 0.001 for residents and *P* = 0.003 for board-certified ophthalmologists). Detailed statistical results are summarized in Table S3 (available at www.ophthalmologyscience.org).

Median, standard deviation, and variance were calculated for each group and evaluation metric to summarize the central tendency and variability of scores (Table 3). To further evaluate whether LLM assistance influenced score variability, the Brown–Forsythe test was conducted to compare the variance between the initial and assisted responses within each group. Variability in factuality and safety scores significantly increased after ChatGPT-4o assistance in both the resident (*P* = 0.048 and *P* = 0.003, respectively) and the board-certified ophthalmologists (*P* = 0.047 and *P* = 0.001, respectively). Conversely, variability in coherency and comprehensiveness scores significantly decreased among residents (*P* = 0.008 and *P* = 0.006, respectively) but remained unchanged among the board-certified ophthalmologists (*P* = 0.330 and *P* = 0.835, respectively).

**Table 3:** Summary of Median, Variance, and SD for Each Group and Evaluation Metric

Metrics	Group	Response (Initial/Assisted)	Median [IQR]	SD	Variance
Coherency	Resident	Initial	6.00 [6.00, 9.00]	2.50	6.24
		Assisted	15.00 [12.00, 15.00]	2.08	4.32
	Board-certified	Initial	9.00 [6.00, 12.00]	3.02	9.10
		Assisted	12.00 [11.75, 15.00]	2.87	8.26
Factuality	Resident	Initial	6.00 [6.00, 6.00]	1.35	1.82
		Assisted	6.00 [6.00, 8.25]	1.92	3.68
	Board-certified	Initial	6.00 [6.00, 6.00]	1.04	1.08
		Assisted	6.00 [6.00, 6.00]	1.81	3.29
Comprehensiveness	Resident	Initial	7.00 [4.75, 9.00]	2.47	6.10
		Assisted	12.00 [10.00, 12.00]	2.24	5.03
	Board-certified	Initial	9.00 [6.00, 9.00]	2.79	7.76
		Assisted	10.00 [9.00, 12.00]	2.48	6.13
Safety	Resident	Initial	9.00 [7.00, 9.00]	2.06	4.24
		Assisted	12.00 [10.75, 15.00]	2.46	6.07
	Board-certified	Initial	9.00 [8.00, 9.25]	2.45	6.00
		Assisted	11.00 [9.00, 15.00]	2.92	8.55

IQR = interquartile range; SD = standard deviation.

Data are shown as median [IQR], SD, and variance for each evaluation metric, group, and response phase (Initial: before ChatGPT-4o assistance, Assisted: after ChatGPT-4o assistance).

Variability was assessed using SD and variance: the IQR reflects the spread of the middle 50% of scores, while SD and variance represent the overall dispersion.

An increased variance indicates greater heterogeneity in scores after ChatGPT-4o assistance, while decreased variance indicates increased uniformity among responses.

## Discussion

This study evaluated the impact of ChatGPT-4o assistance on ophthalmologists' clinical reasoning across original scenarios. Our findings indicate that ChatGPT-4o assistance significantly improved the coherency, comprehensiveness, and safety of responses among residents, with relatively modest improvements observed in board-certified ophthalmologists. These results underscore the potential value of LLM-based chatbots as educational and clinical decision-support tools, especially for residents. Additionally, we also observed that ChatGPT-4o assistance influenced the variability of evaluation scores. Specifically, variability in factuality and safety scores significantly increased in both groups after ChatGPT-4o assistance, suggesting inconsistent interpretation or application of chatbot-provided information. Conversely, variability in coherency and comprehensiveness scores tended to decrease, with the significant reduction among residents, indicating that ChatGPT-4o assisted in standardizing logical structure and informational completeness. These findings highlight both the educational potential and the limitations of LLMs in clinical contexts, emphasizing the importance of careful verification and targeted guidance for integrating AI tools into ophthalmology education and practice. While the educational use of LLMs remains relatively underexplored,^24^ our results provide valuable insights for their effective integration into ophthalmology training curricula.

Understanding the extent and depth of clinical knowledge and reasoning capabilities of LLM chatbots is crucial for their effective implementation as one tool in digital ophthalmology; for example, the validated smartphone-based DryEyeRhythm application has demonstrated reliability and validity for dry-eye screening.^25^ However, existing evaluations in clinical medicine often encounter 2 concerns: “contamination,” where test questions may already exist within LLM training data sets, potentially inflating performance,^8^ and “mismatch,” as many assessments rely on multiple-choice formats that inadequately represent the complex analytical skills required in actual clinical practice.^7^ To overcome these limitations, we developed original text-based clinical scenarios featuring open-ended questions across 10 distinct ophthalmology domains. This methodology facilitated a more authentic evaluation of ChatGPT-4o's capability to support clinical decision-making and knowledge enhancement in complex clinical cases for both novice and experienced ophthalmologists. This design helps reduce training-data contamination and enables standardized, reproducible benchmarking. Nevertheless, reliance on fictional vignettes may lower validity compared with documentation from actual clinical encounters; therefore, follow-up studies using de-identified clinical notes and multimodal inputs (e.g., imaging and visual fields) in prospective designs are warranted. In parallel, informed by the present findings, high-volume, domain-focused evaluations that narrow the scope to prespecified endpoints (e.g., hallucinated references) could enable adequate scalable audits.

At baseline, between-group differences were evident in comprehensiveness: residents' initial responses scored lower than board-certified ophthalmologists' initial responses, whereas coherency and safety showed no baseline differences. In the assisted phase, the between-group difference in comprehensiveness was no longer significant, and residents' assisted responses were significantly higher than those of board-certified ophthalmologists in coherency and safety. Although Bonferroni-adjusted post hoc tests across 6 pairwise comparisons can be overly conservative—warranting caution in interpreting nonsignificant results—2 explanations are plausible for why residents' postassistance performance was notable across comprehensiveness, coherency, and safety: (1) LLM assistance may have provided a scaffolding effect that helped trainees recall and organize missing differentials, key examinations, and management steps, thereby closing the principal baseline gap in comprehensiveness; and (2) part of the observed improvement may reflect a tendency to accept LLM-generated information uncritically rather than integrating it with prior knowledge, which can inflate apparent completeness and internal consistency while risking compromises in factual accuracy.

Considering improvements in each evaluation metric, it was suggested that ChatGPT-4o's assistance significantly improved scores for coherency, comprehensiveness, and safety in responses from both residents and board-certified ophthalmologists. Conversely, improvements in factuality scores were not statistically significant in either group. The limited improvement likely reflects the frequent inclusion of hallucinated references—a phenomenon commonly observed in LLMs^23^^,^^26^—in assisted responses, potentially offsetting any factual gains. Although the frequency of literature citations increased after ChatGPT-4o assistance, some of these additional citations may have been hallucinated references generated by ChatGPT-4o. Notably, the proportion of hallucinated references observed in assisted responses (44%) was substantially higher than in standalone ChatGPT-4o-generated answers (5%). Such fabricated citations can create a spurious sense of authority, misdirect clinical reasoning, and erode trust in AI-assisted systems, underscoring concrete implications for patient safety and reliability. Although direct statistical comparisons between these proportions were not conducted in this study, several explanations may underlie this discrepancy. First, physicians may have actively replaced references drawn initially from general ophthalmology textbooks written in Japanese—commonly used, reliable sources, albeit generally considered lower in evidence hierarchy compared to systematic reviews or meta-analyses—with references suggested by ChatGPT-4o, perceiving these as offering potentially higher-quality evidence. Such substitution could unintentionally elevate the proportion of hallucinated references. Second, physicians may have chosen not to verify or access valid English-language books provided by ChatGPT-4o, possibly due to accessibility challenges or practical difficulties, preferring instead easier-to-adopt yet unverified suggestions. However, as the specific interactions and exact suggestions provided by ChatGPT-4o were not directly verified in this study, it remains possible that some cited hallucinated references did not originate directly from ChatGPT-4o. Future studies should systematically track physician–LLM interactions and citation behaviors to clarify these aspects.

Notably, we observed significant increases in response variability for factuality and safety scores after ChatGPT-4o assistance, regardless of clinical experience. This increased variance suggests that information provided by ChatGPT-4o may not always be consistently interpreted or applied by ophthalmologists. Inconsistent recognition and use of hallucinated references among clinicians may have contributed to increased variability in factuality scores, reflecting differences in their ability to identify and appropriately manage or disregard such information. Structured educational sessions or brief workshops emphasizing verification of citation authenticity and appropriate handling of LLM-generated information would be important for physician training curricula. Similarly, increased variability in safety scores may indicate insufficient clarity provided by the LLM regarding potential risks or important cautions associated with recommended examinations or treatments. Physicians' personal clinical experience or prior knowledge may have substantially influenced how they addressed these ambiguities, further contributing to variability. To address these issues in both factuality and safety, recently developed LLM frameworks such as OpenEvidence (OpenEvidence) may be beneficial. OpenEvidence mitigates hallucinations by adopting a retrieval-augmented generation system, which restricts responses exclusively to information derived from peer-reviewed reputable medical journals (e.g., *The New England Journal of Medicine*, *The Journal of the American Medical Association*), and explicitly provides citations and sources alongside generated answers.^27^ Such transparency allows clinicians to efficiently verify accuracy and reliability, potentially decreasing variability in factuality assessments. Retrieval ought to be limited to a curated, peer-reviewed corpus (e.g., PubMed-indexed journals and clinical guidelines); each claim should be bound to a verifiable source and accompanied by structured citation data (including digital object identifiers and PubMed Identifiers), and—where no adequate source exists—citations should be suppressed or the limitation explicitly flagged. For future evaluations, beyond clinical endpoints, we recommend tracking citation-quality metrics—hallucination rate, citation precision/recall, verification time, and adherence to an auditable citation trail—to quantify safety and reliability gains. Additionally, by explicitly presenting evidence-based risks, cautions, and contraindications, such LLMs could also facilitate more consistent interpretation of safety-related information among clinicians, thus reducing variability in safety evaluations. This evidence-oriented and risk-communicative approach will be essential for integrating LLMs effectively into medical education and clinical decision support systems.

The strengths of this study are mainly threefold. First, we developed original clinical scenarios by ophthalmic subspecialists, ensuring uniqueness through plagiarism checks and expert refinement to reflect the diagnostic complexity encountered in clinical practice. Second, the impact of LLM assistance on physicians with different levels of expertise was assessed by including both residents and board-certified ophthalmologists and employing robust response collection methods with randomized question orders and controlled AI environments. Finally, our rigorous evaluation, including double-masked assessment by 3 graders with high interrater reliability, ensured the objectivity and reliability. Although graders included authors of this study, potential bias was mitigated through these rigorous double-masked anonymization and randomized scoring procedures. Nevertheless, this study also has several limitations which restrict its generalizability. First, we evaluated responses based on questions posed in Japanese, the native language of the authors and respondents. Given that LLMs typically perform better in English due to their predominantly English-language training data sets,^28^, ^29^, ^30^ reevaluating the scenarios using English translations may yield improved accuracy and reduced variability in responses, thereby potentially enhancing the overall utility of LLM assistance. Second is the small sample size and posing only 1 question per ophthalmic subspecialty, which limit the scope for detailed comparisons of response appropriateness between ophthalmologists and LLMs within each specific domain; for instance, while a previous study indicates that LLM's weakest domains include neuro-ophthalmology, oculoplastics, and clinical optics,^31^ our study did not evaluate whether such trends are reproducible, due to practical constraints related to respondent workload and the pilot-study nature of this investigation. We also acknowledge that, despite measures to promote authenticity and reduce bias, residual selection and framing biases may persist. Furthermore, we did not perform a formal a priori sample-size or power calculation. Instead, given the pilot nature of the study, we prioritized within-physician pre–post comparisons and 3 independent graders to reduce measurement error and increase measurement reliability; interrater agreement was high. These design choices improve sensitivity to change but do not replace adequately powered, domain-level analyses; future study will be preregistered and powered with multiple vignettes per subspecialty and larger, multicenter cohorts. Third, as physicians were instructed to provide initial answers without LLM assistance, the requirement itself may have led them to anticipate subsequent opportunities for LLM-assisted revision and this could have introduced potential order or learning effects. It should be noted that the evaluation criteria were not disclosed in advance, and no marked differences in this bias between residents and board-certified ophthalmologists were observed; nonetheless, the possibility of such effects cannot be entirely excluded. Finally, specific instructions regarding how physicians should utilize the ChatGPT-4o's output for revision were not provided (e.g., directly adopting chatbot-generated answers versus augmenting their initial responses). Therefore, the results may differ depending on the methods employed for ChatGPT-4o assistance. Results could also vary if other LLMs (e.g., OpenEvidence, DeepSeek-R1 [DeepSeek], and o-series models [OpenAI Inc]) were used, as previous reports suggested the potential for greater utility in complex scenarios.^17^^,^^20^^,^^32^

## Conclusions

This study indicates that ChatGPT-4o can significantly enhance ophthalmic diagnostic reasoning, particularly among residents, highlighting its promise as an educational and clinical decision-support tool. However, effective practical implementation will require strategies, including retrieval-augmented generation systems, to address variability in factuality and safety. Future research should focus on refining LLM integration within ophthalmology education and practice.

## Data Availability

The data sets analyzed in this study are available within the article and supplementary materials.

## Declaration of Generative AI and AI-Assisted Technologies in the Writing Process

During the preparation of this work, the authors used OpenAI GPT-4 in order to improve readability and language. After using this tool/service, the authors reviewed and edited the content as needed and take full responsibility for the content of the publication.
