# Supplementary material for: Evolving Consultation: Enhancing Ophthalmic Diagnostic Performance Using Large Language Model
Source: Ophthalmol Sci. 2025 Nov 11;6(2):101004. doi: 10.1016/j.xops.2025.101004 (PMC12919258; doi:10.1016/j.xops.2025.101004)
Supplement: Supplemental Component 1 [file mmc1.pdf]

The Actual Native Japanese Scenarios Used in This Study

【症例 1】
72 歳の男性。数年前より徐々に進行する右眼球突出を主訴に当院受診となった。既往歴として前立腺がん(肺転移あり)、糖尿病、高脂血症がある。矯正視力両眼 1.0、眼圧は右 25 mmHg で左 17 mmHg、眼内に異常所見は認めない。中心フリッカ値は両眼ともに 40 Hz、HESS 赤緑試験で内転下転制限を認めた。眼球突出度は右 25 mm、左 15 mm であった。

【症例 2】
55 歳の男性。数日前からの右眼の痛みと視力低下を主訴に受診した。既往歴としては数年前に内科にて糖尿病・高脂血症の指摘はあったが、現在通院していない。眼の既往歴としては両白内障手術歴がある。
矯正視力は右 0.04 で左 0.9、眼圧は右 44 mmHg で左 19 mmHg。右眼の前房は深いが角膜浮腫のため観察しづらく、前房内炎症の有無は確認困難で、同様に眼底も透見不良だった。

【症例 3】
5 歳男児。3 歳児検診で視力不良を指摘され近医眼科を受診後、眼鏡矯正を開始して経過を見ていたが矯正視力が 1.0 まで出ないため、原因精査として当院に紹介となった。
矯正視力は右 0.6 左 0.5 であった。眼圧は右 14 mmHg で左 12 mmHg だった。前眼部・中間透光体に異常を認めなかった。眼底は豹紋状眼底を認めるがその他明らかな変性所見は無かった。全視野網膜電図を施行したところ混合応答では a 波に比して b 波の波形は小さく、錐体応答では幅広の a 波が検出された。
弟の 3 歳男児も同様に近視矯正をしているがやはり視力不良があり精査予定である。

【症例 4】
42 歳、東南アジア人女性。2 週間ほど前から右眼の歪みを自覚し近医を受診、精査目的に当院紹介となった。喫煙歴なし。小児期から近視があり、現在は右-7.0 D、左-6.0 D のソフトコンタクトレンズを使用している。矯正視力は右 0.5、左 1.0、眼圧は右 14 mmHg、左 16 mmHg であった。前眼部および中間透光体に異常はない。眼底検査で両眼に豹紋状変化があり、後極部に小さな黄白色点状病変が散在している。右眼黄斑部にはわずかな出血を伴っており、一致して光干渉断層計で網膜下に辺縁不整な隆起性病変がみられた。

【症例 5】
40 代男性。昨日から「なんとなく見えにくい感じがある」という主訴で近医受診、精査加療目的に当院紹介となった。眼に関する既往歴としては、1 ヶ月前に両眼でエキシマレーザー角膜屈折矯正(レーシック)手術の既往、20 年前にどちらかの眼のぶどう膜炎の既往(詳細不明、ステロイド点眼で軽快したとのこと)がある。
近医受診翌日に当院受診し、矯正視力は右 0.4、左 1.2、眼圧は右 5 mmHg、左 8 mmHg であった。中心フリッカ値は右 22 Hz、左 40 Hz、対光反射は両眼で迅速であったが、右眼の相対性求心性瞳孔反応欠損(relative afferent pupillary defect)が陽性であった。細隙灯顕微鏡検査では前房は深く清明、角膜も清明で、特記すべき異常を認めない。

【症例 6】
30 歳、男性。2 週間前より右眼の充血と痛みがあり近医眼科を受診した。抗菌薬の点眼を処方されるも症状は改善せず、視力も低下してきたため当院に受診した。ソフトコンタクトレンズ装用者で、ヘルペス角膜炎の既往がある。
矯正視力は右 0.1、左 1.2 で、眼圧は右 28 mmHg で左 14 mmHg であった。細隙灯顕微鏡検査では結膜充血と毛様充血に加え、角膜中央に円形の浸潤とそれを中心とした浮腫を認め、浸潤部位の内皮面に角膜後面沈着物を認めた。角膜上皮は粗造であるものの明らかな上皮欠損は認めなかった。強い痛みを訴えており、それ以上の眼底検査などは困難であった。

【症例 7】
2 歳女児。右の瞳孔が白く見えることに親が気付き、当院に受診した。これまでの発達に異常の指摘はなく、特に眼疾患の家族歴は認めない。左眼を遮蔽すると嫌悪反射を認めた。眼圧は右 12 mmHg、左 12 mmHg であった。前眼部に異常は認めなかった。散瞳後の検査では、右眼硝子体中には白色の乳頭大の腫瘤性病変が多数浮遊しており、眼底所見ははっきり観察できなかった。

【症例 8】

68 歳の男性。糖尿病、高脂血症、高血圧の既往がある。最近眼鏡が合わなくなっており、特に右眼は視力低下と眼痛が強いということで当院眼科を受診した。視力は右 0.03 (n.c.) で左 0.2 (1.2×K.B.×S -1.5D)、眼圧は右 46 mmHg、左 16 mmHg だった。細隙灯顕微鏡検査では右眼に角膜浮腫ならびに虹彩ルベオーシス、軽度白内障を認めたが、左眼には軽度白内障以外に大きな異常所見を認めなかった。また、眼底所見として右眼は複数の軟性白斑と斑状出血を伴っていたが、左眼ではごくわずかな点状出血と毛細血管瘤が確認された。

【症例 9】

65 歳、女性。数ヶ月前からものが 2 重に見えることに気づき近医眼科を受診した。両眼複視の訴えがあり、斜視による症状を疑われ、精査加療目的に当院紹介となった。既往歴として高血圧、糖尿病、帯状疱疹がある。
矯正視力は右 1.0、左 1.0 で、眼圧は右 15mmHg、左 17mmHg だった。細隙灯顕微鏡検査・眼底検査では異常を認めなかった。眼位は他覚的定量眼位検査(alternate prism cover test)では近見 3△R/L、遠見 6△ET+8△R/L であり、むき運動で左眼のわずかな上転障害を認めた。近見での複視の訴えはないが遠見で複視の訴えがあった。

【症例 10】

27 歳、男性。1 か月ほど前からかすんで見えることがあり、眼科受診となった。特に既往歴の指摘はない。
矯正視力は右 1.0、左 1.0 で、眼圧は右 17 mmHg、左 16 mmHg だった。細隙灯顕微鏡検査で両眼に毛様充血と前房内の炎症細胞を認め、粗大な角膜後面沈着物もわずかに認めた。眼底検査で硝子体混濁があるも、網膜血管の視認性は問題なかった。光干渉断層計では黄斑浮腫は認めず、視神経乳頭がわずかに腫脹していた。対光反射は両眼とも迅速で、中心フリッカ値は右 42 Hz、左 43 Hz であった。また両眼とも相対性求心性瞳孔反応欠損(relative afferent pupillary defect)は陰性であった。

Translations of the Scenarios into English

【Case 1】

A 72-year-old male patient presented to our hospital with a chief complaint of gradually progressing right eye protrusion over the past few years. His medical history included prostate cancer (with lung metastasis), diabetes, and hyperlipidemia. Corrected visual acuity was 1.0 in both eyes, intraocular pressure was 25 mmHg in the right eye and 17 mmHg in the left eye, and no abnormal findings were observed in either eye. Central flicker threshold was 40 Hz in both eyes, and the HESS red-green test revealed restriction of adduction and downward movement. Proptosis was 25 mm in the right eye and 15 mm in the left eye.

【Case 2】

A 55-year-old male presented to the ophthalmology department with complaints of right eye pain and decreased vision that had begun several days prior. His past medical history included a diagnosis of diabetes and hyperlipidemia by an internist several years ago, but he has since dropped out of treatment. His ophthalmic history includes cataract surgery in both eyes.

Corrected visual acuity was 0.04 in the right eye and 0.9 in the left eye. Intraocular pressure was 44 mmHg in the right eye and 19 mmHg in the left eye. The anterior chamber of the right eye was deep, but corneal edema made observation difficult, and the presence of intraocular inflammation could not be confirmed. Similarly, the fundus was poorly visible.

【Case 3】

A 5-year-old boy was noted to have poor vision during a 3-year-old health checkup and visited a local ophthalmologist. Glasses were prescribed and his visual acuity was monitored. However, as his corrected visual acuity did not reach 1.0 decimal unit, he was referred to our hospital for further evaluation.

His corrected vision was 0.6 in the right eye and 0.5 in the left eye. Intraocular pressure was 14 mmHg in the right eye and 12 mmHg in the left eye. No abnormalities were noted in the anterior segment or intermediate transparent body. The fundus showed tigroid fundus but no other obvious degenerative findings. A full-field electroretinogram was performed, revealing that in the mixed response, the b-wave was smaller than the a-wave, and in the cone response, a square a-wave was detected.

His 3-year-old brother is also undergoing myopia correction but has poor vision and is scheduled for further evaluation.

【Case 4】

A 42-year-old Southeast Asian female. She noticed metamorphopsia in her right eye about two weeks ago and visited a local ophthalmologist and was referred to our hospital for further examination. She has no history of smoking. She has had myopia since childhood and currently wears soft contact lenses with a prescription of -7.0 D in her right eye and -6.0 D in her left eye. Her corrected visual acuity was 0.5 in her right eye and 1.0 in her left eye, and her intraocular pressure was 14 mmHg in her right eye and 16 mmHg in her left eye. No abnormalities were noted in the anterior segment or intermediate transparent media. Fundus examination revealed tigroid fundus in both eyes with small yellowish-white punctate lesions scattered in the posterior pole. The right macula showed minimal hemorrhage, and corresponding to this, optical coherence tomography revealed an irregularly shaped elevated lesion beneath the retina.

【Case 5】

A 40-year-old male patient visited a local clinic yesterday complaining of "somewhat blurred vision" and was referred to our hospital for further examination and treatment. His ocular history includes bilateral excimer laser keratectomy (LASIK) one month ago and uveitis in one eye 20 years ago (details unknown, but reportedly resolved with steroid eye drops).

He visited our hospital the day after his initial consultation, with corrected visual acuity of 0.4 in the right eye and 1.2 in the left eye, and intraocular pressure of 5 mmHg in the right eye and 8 mmHg in the left eye. The central flicker threshold was 22 Hz in the right eye and 40 Hz in the left eye. The pupillary light reflex was rapid in both eyes, but the right eye showed a positive relative afferent pupillary defect (RAPD). Slit-lamp microscopy revealed a deep and clear anterior chamber, a clear cornea, and no notable abnormalities.

【Case 6】

30-year-old male. Two weeks ago, he experienced redness and pain in his right eye and visited a local ophthalmologist. He was prescribed antibiotic eye drops; however, his symptoms did not improve, and his visual acuity began to deteriorate, therefore he visited our hospital. He usually wears soft contact lenses and has a history of herpes keratitis.

Corrected visual acuity was 0.1 in the right eye and 1.2 in the left eye, with intraocular pressure of 28 mmHg in the right eye and 14 mmHg in the left eye. Slit-lamp microscopy revealed conjunctival redness and ciliary redness, along with a circular infiltrate in the central cornea and edema centered around it. Posterior corneal deposits were observed on the endothelial surface of the infiltrate. The corneal epithelium was rough but no obvious epithelial defects were noted. The patient reported severe pain, making further fundus examination difficult.

【Case 7】

A 2-year-old girl was brought to our hospital by her parents, who noticed that her right pupil appeared white. There were no reports of developmental abnormalities, and there was no family history of eye disease. When the left eye was covered, an aversion response was observed. Intraocular pressure was 12 mmHg in both eyes. No abnormalities were observed in the anterior segment. Following dilation, examination revealed multiple white, optic disc-sized tumor-like lesions floating in the vitreous humor of the right eye, and the fundus findings could not be clearly observed.

【Case 8】

A 68-year-old male. He has a history of diabetes, hyperlipidemia, and hypertension. Recently, his glasses have become ill-fitting, and he visited our ophthalmology department complaining of severe vision loss and eye pain, particularly in his right eye.

Visual acuity was 0.03 (n.c.) in the right eye and 0.2 (1.2×K.B.×S -1.5D) in the left eye. Intraocular pressure was 46 mmHg in the right eye and 16 mmHg in the left eye. Slit-lamp microscopy revealed corneal edema, iris rubeosis, and mild cataracts in the right eye, but no significant abnormalities other than mild cataracts were observed in the left eye. Fundus examination showed multiple soft white spots and patchy hemorrhages in the right eye, while only minimal punctate hemorrhages and microaneurysms were noted in the left eye.

【Case 9】

65-year-old female. She noticed double vision several months ago and visited her local ophthalmologist. She complained of double vision in both eyes, and symptoms of strabismus were suspected. She was referred to our hospital for further examination and treatment. Her medical history includes hypertension, diabetes, and herpes zoster.

Her corrected visual acuity was 1.0 in both eyes, and her intraocular pressure was 15 mmHg in the right eye and 17 mmHg in the left eye. No abnormalities were detected on slit-lamp microscopy or fundus examination. Eye position was assessed using the alternate prism cover test, showing 3 prism diopters of right/left for near vision and 6 prism diopters of esotropia plus 8 prism diopters of right/left for distance vision. A slight upward deviation of the left eye was noted during eye movement. She did not report double vision during near vision but did report it during distance vision.

【Case 10】

27-year-old male. He visited an ophthalmologist about a month ago because he had been experiencing blurred vision. He had no particular medical history.

Corrected visual acuity was 1.0 in both eyes, and intraocular pressure was 17 mmHg in the right eye and 16 mmHg in the left eye. Slit-lamp microscopy revealed ciliary congestion and inflammatory cells in the anterior chamber of both eyes, as well as minimal keratic precipitates. Fundus examination showed vitreous opacity, but retinal vessel visibility was normal. Optical coherence tomography showed no macular edema; however, the optic nerve head was slightly swollen. The pupillary light reflex was rapid in both eyes, with a central flicker threshold of 42 Hz in the right eye and 43 Hz in the left eye. Additionally, both eyes showed no relative afferent pupillary defect (RAPD).
