# Supplementary material for: Evolving Consultation: Enhancing Ophthalmic Diagnostic Performance Using Large Language Model
Source: Ophthalmol Sci. 2025 Nov 11;6(2):101004. doi: 10.1016/j.xops.2025.101004 (PMC12919258; doi:10.1016/j.xops.2025.101004)
Supplement: Supplemental Component 2 [file mmc2.pdf]

Author's note—Abbreviations and units seen in the responses. Unless otherwise specified, the following interpretations and spellings are used throughout this supplement: acetylcholine receptor (AChR), alternate prism cover test (APCT), branching vascular network (BVN), central serous chorioretinopathy (CSC), choroidal neovascularization (CNV), computed tomography (CT), fluorescein angiography (FA), Hess red-green test (Hess), indocyanine green angiography (ICGA), magnetic resonance imaging (MRI), multifocal pattern dystrophy simulating fundus flavimaculatus (MPDSFF; appears as "MPPEH" in some responses), myopic choroidal neovascularization (mCNV), optical coherence tomography (OCT), optical coherence tomography angiography (OCTA), pachychoroid neovasculopathy (PNV), polypoidal choroidal vasculopathy (PCV), prism diopters (Δ), relative afferent pupillary defect (RAPD), retinal pigment epithelium (RPE), vascular endothelial growth factor (VEGF)

The Actual Native Japanese Representative Responses

【症例】
65 歳、女性。数ヶ月前からものが 2 重に見えることに気づき近医眼科を受診した。両眼複視の訴えがあり、斜視による症状を疑われ、精査加療目的に当院紹介となった。既往歴として高血圧、糖尿病、帯状疱疹がある。
矯正視力は右 1.0、左 1.0 で、眼圧は右 15mmHg、左 17mmHg だった。細隙灯顕微鏡検査・眼底検査では異常を認めなかった。眼位は他覚的定量眼位検査(alternate prism cover test)では近見 3△R/L、遠見 6△ET+8△R/L であり、むき運動で左眼のわずかな上転障害を認めた。近見での複視の訴えはないが遠見で複視の訴えがあった。

【質問】
あなたは眼科医で、提示されたシナリオ症例の診療情報を得たとします。
この場合、最も可能性の高い臨床診断についてその根拠を明示しながら 3 つ程度挙げて、さらに追加して行うべき検査および対応も優先度の高いものから 3 つ程度挙げてください。追加して行うべき検査および対応については、まとめて記載で構いません。
必要であれば、回答の根拠を文献からの引用で裏付けながら記載してください。文献についてはホームページのリンクを記載するか、雑誌もしくは書籍であれば代表著者、雑誌名、出版年を明記してください。
回答は常体(である調)としてまとめてください。
なお、必要に応じてウェブ検索を行っても構いません。

専門医 A の場合

1. Initial Response と評価者 B によるスコア:

高齢者の後天性の軽度の上下斜視を伴う、内斜視であり、遠見時に複視を強く自覚することから病歴からは Sagging eye syndrome を疑う。単独の脳神経の障害では左眼の上転障害や内斜視を一元的には説明しづらい。鑑別すべき疾患として、中枢神経疾患や重症筋無力症などが存在する。

必要な検査としては、頭部 MRI や頭部 CT など、頭蓋内疾患の検索および上直筋と外直筋を結ぶ靭帯の断裂の確認、重症筋無力症の鑑別のための問診の追加および抗アセチルコリン受容体抗体の測定、HESS 赤緑試験による眼球運動異常の定量などである。https://eyewiki.org/Sagging_Eye_Syndrome

上記により他疾患の除外が行われた場合の対応としてはプリズム眼鏡の処方や患者が希望する場合には斜視手術による複視治療を行う。

評価者 B によるスコア				
一貫性 (Coherency)	事実性 (Factuality)	包括性 (Comprehensiveness)	安全性 (Safety)	その他コメント(適宜)
5	3	4	5	頭部 MRI での動脈瘤除外は安全性への配慮が高いと考えた

2. ChatGPT-4o に問いかけた Prompt:

【症例】

65 歳、女性。数ヶ月前からものが 2 重に見えることに気づき近医眼科を受診した。両眼複視の訴えがあり、斜視による症状を疑われ、精査加療目的に当院紹介となった。既往歴として高血圧、糖尿病、帯状疱疹がある。

矯正視力は右 1.0、左 1.0 で、眼圧は右 15mmHg、左 17mmHg だった。細隙灯顕微鏡検査・眼底検査では異常を認めなかった。眼位は他覚的定量眼位検査 (alternate prism cover test)では近見 3△R/L、遠見 6△ET+8△R/L であり、むき運動で左眼のわずかな上転障害を認めた。近見での複視の訴えはないが遠見で複視の訴えがあった。

【質問】

あなたは眼科医で、提示されたシナリオ症例の診療情報を得たとします。

この場合、最も可能性の高い臨床診断についてその根拠を明示しながら 3 つ程度挙げて、さらに追加して行うべき検査および対応も優先度の高いものから 3 つ程度挙げてください。追加して行うべき検査および対応については、まとめて記載で構いません。

必要であれば、回答の根拠を文献からの引用で裏付けながら記載してください。文献についてはホームページのリンクを記載するか、雑誌もしくは書籍であれば代表著者、雑誌名、出版年を明記してください。
回答は常体(である調)としてまとめてください。
なお、必要に応じてウェブ検索を行っても構いません。
上記の問いに対して下記のように考えました。
高齢者の後天性の軽度の上下斜視を伴う、内斜視であり、遠見時に複視を強く自覚することから病歴からは Sagging eye syndrome を疑う。単独の脳神経の障害では左眼の上転障害や内斜視を一元的には説明しづらい。鑑別すべき疾患として、中枢神経疾患や重症筋無力症などが存在する。
必要な検査としては、頭部 MRI や頭部 CT など、頭蓋内疾患の検索および上直筋と外直筋を結ぶ靭帯の断裂の確認、重症筋無力症の鑑別のための問診の追加および抗アセチルコリン受容体抗体の測定、HESS 赤緑試験による眼球運動異常の定量などである。https://eyewiki.org/Sagging_Eye_Syndrome
上記により他疾患の除外が行われた場合の対応としてはプリズム眼鏡の処方や患者が希望する場合には斜視手術による複視治療を行う。
この答えを修正してください。

3. Assisted Response と評価者 B によるスコア:
上記症例は Sagging eye syndrome（SES）を疑う。
根拠として
・高齢者（65 歳）であること。SES は加齢に伴う結膜・支持組織の緩み（結膜弛緩症や眼球周囲支持組織の退行変性）により生じるため、高齢者に好発する。
・軽度の眼位異常（遠見で 6△ET+8△R/L）を認めていること。SES では遠見での内斜視（特に加齢内斜視）が典型的である。
・斜視による両眼複視の訴えがあり、特に遠見で複視を自覚している点も SES と一致する。
・眼球運動異常は軽度で、左眼のわずかな上転障害があるが、これも眼球運動制限が強くない点で SES に合致する。

■矛盾する所見
・左眼のわずかな上転障害がある点は SES の典型所見ではない。SES は通常、眼球運動制限を伴わず、支持組織の弛緩による眼位のズレが主体であるため、明確な眼筋麻痺様の運動障害は通常認めない。

・既往に糖尿病・高血圧があることは、微小血管障害による動眼神経麻痺や眼筋麻痺を示唆するため、SES 以外の神経筋疾患の鑑別が必要となる。
・複視が遠見に限局しているが、神経麻痺など他の原因でも遠見複視を呈することがあり、単純に SES とは断定できない。

■鑑別疾患
・動眼神経麻痺（糖尿病性動眼神経麻痺）: 糖尿病患者に多く、片眼の眼球運動障害と複視を呈する。特に上転障害がある場合は動眼神経障害を疑う。
・老年性内斜視（加齢内斜視）: SES と類似するが、SES は眼窩支持組織の構造的変化が明らかであるのに対し、老年性内斜視は支持組織変化が必ずしも関与しない。
・重症筋無力症 : 眼筋の疲労性麻痺による複視。眼球運動制限や複視の程度が日内変動することが多い。
・甲状腺眼症 : 眼筋肥大や炎症による眼球運動障害、複視が生じるが、眼球突出や眼瞼異常が伴うことが多い。
・脳幹疾患や動眼神経核病変 : 眼球運動障害や複視を呈し、神経症状を伴うことがある。

■必要な検査
・頭部・眼窩 MRI 検査 : 眼窩支持組織の状態や眼筋の異常（肥大、萎縮、炎症）を評価し、SES の診断補助や中枢性神経障害の除外に用いる。
・眼筋機能の詳細検査 : 眼球運動検査（動画撮影や HESS 赤緑試験）、眼位検査の繰り返し。
・神経学的評価 : 糖尿病性神経障害の有無、動眼神経麻痺の評価。
・血液検査 : 糖尿病コントロール状況の把握、甲状腺機能検査（甲状腺眼症の除外）重症筋無力症の診断（抗アセチルコリン受容体抗体、抗筋特異的キナーゼ抗体の測定）。
・眼筋電図や筋機能検査（必要に応じて）: 重症筋無力症の評価に用いる場合がある。

評価者 B によるスコア				
一貫性 (Coherency)	事実性 (Factuality)	包括性 (Comprehensiveness)	安全性 (Safety)	その他コメント(適宜)
5	2	5	4	概ね記載がされている。頭部 MRI の緊急性についての言及もあるとよいと考えた

レジデント C の場合

1. Initial Response と評価者 B によるスコア:

a. 臨床診断:眼部帯状疱疹
根拠:糖尿病　帯状疱疹　複視

b. 臨床診断:Sagging eye syndrome
根拠:斜視　遠見 6△ET+8△R/L 遠見で複視の訴え

c. 臨床診断:麻痺性斜視
数ヶ月前からの複視　上転障害

追加検査、対応
頭部 CT、頭部 MRI、MRA
血液検査で水痘帯状疱疹ウイルス抗体の測定

評価者 B によるスコア				
一貫性 (Coherency)	事実性 (Factuality)	包括性 (Comprehensiveness)	安全性 (Safety)	その他コメント(適宜)
2	2	2	3	上斜筋麻痺および動眼神経麻痺についての言及もあるとよいと考えた。

2. ChatGPT-4o に問いかけた Prompt:

【症例】
65 歳、女性。数ヶ月前からものが 2 重に見えることに気づき近医眼科を受診した。両眼複視の訴えがあり、斜視による症状を疑われ、精査加療目的に当院紹介となった。既往歴として高血圧、糖尿病、帯状疱疹がある。
矯正視力は右 1.0、左 1.0 で、眼圧は右 15mmHg、左 17mmHg だった。細隙灯顕微鏡検査・眼底検査では異常を認めなかった。眼位は他覚的定量眼位検査(alternate prism cover test)では近見 3△R/L、遠見 6△ET+8△R/L であり、むき運動で左眼のわずかな上転障害を認めた。近見での複視の訴えはないが遠見で複視の訴えがあった。
【質問】
あなたは眼科医で、提示されたシナリオ症例の診療情報を得たとします。
この場合、最も可能性の高い臨床診断についてその根拠を明示しながら 3 つ程度挙げて、さらに追加して行うべき検査および対応も優先度の高いものから 3

つ程度挙げてください。追加して行うべき検査および対応については、まとめて記載で構いません。
必要であれば、回答の根拠を文献からの引用で裏付けながら記載してください。文献についてはホームページのリンクを記載するか、雑誌もしくは書籍であれば代表著者、雑誌名、出版年を明記してください。
回答は常体(である調)としてまとめてください。
なお、必要に応じてウェブ検索を行っても構いません。

3. Assisted Response と評価者 B によるスコア:
【臨床診断の鑑別（可能性の高いもの 3 つ）】
a. 左上斜筋麻痺（左滑車神経麻痺、Fourth nerve palsy）
　最も可能性が高い診断である。遠見で複視が目立ち、近見で軽減するという所見、斜視角が遠見で増大（6△ET+8△R/L）し、左眼のわずかな上転障害がある点から、左上斜筋の機能低下が疑われる。上斜筋は下外転を主な作用とするため、障害があると垂直性および回旋性の複視を訴えることがある。また、加齢による滑車神経麻痺は比較的多く、糖尿病や高血圧などの血管危険因子を持つ本症例では虚血性病因も鑑別に挙がる。

b. 慢性進行性外眼筋麻痺（Chronic Progressive External Ophthalmoplegia：CPEO）
　眼位の異常と上方注視障害があるが、進行が数ヶ月単位と緩徐であり、視力に影響なく、眼瞼下垂などを伴っていない点から完全な像ではないが、鑑別に入る。特に帯状疱疹後に外眼筋障害が報告されている点にも注意が必要である（Zoster ophthalmicus 関連）。

c. ミトコンドリア病（特に Kearns-Sayre 症候群など）
　65 歳とやや高齢であるため典型例ではないが、慢性外眼筋麻痺を含む症候群として、進行性の眼球運動障害の鑑別として考慮する。糖尿病との合併や、他神経症状の出現があればより疑う。

【追加して行うべき検査・対応（優先度順に 3 つ）】
a. Hess 赤緑試験または Lancaster 検査
　眼球運動障害の定量評価に有用で、どの筋が障害されているかを視覚的に明らかにできる。特に上斜筋麻痺では、特徴的な Hess チャート所見が得られる（患側で上斜筋領域が狭く、健側で対側下直筋領域が広がる）。

b. 頭部 MRI 検査（特に脳幹～滑車神経走行領域の評価）

器質的原因（脳幹部梗塞、腫瘍、動脈瘤など）の除外のために画像診断が必要。滑車神経は脳幹背側から出て交叉するため、小病変でも麻痺を起こしうる。血管危険因子があるため虚血性病変の評価も重要。

c. 眼球運動のビデオ記録・筋電図や抗 AChR 抗体（必要に応じて）

重症筋無力症などの他神経筋疾患との鑑別のため。特に眼位変化が日内変動する場合や、複数筋の交互性麻痺を示す場合に有用。症例からは現時点で典型的ではないが、鑑別に入れるべき。

【参考文献】

● Rosenbaum AL, Santiago AP. Clinical Strabismus Management: Principles and Surgical Techniques. W.B. Saunders Company, 1999.

● Walsh FB, Hoyt WF. Clinical Neuro-ophthalmology. Williams & Wilkins, 5th ed, 1998.

● 日本神経眼科学会編『神経眼科学』南江堂, 2013.*(※著者注: この文献は実在しない hallucinated reference である)*

● American Academy of Ophthalmology. Basic and Clinical Science Course (BCSC): Neuro-Ophthalmology. 2023-2024 edition. https://www.aao.org/

必要があれば、各疾患ごとにさらに詳しい診断基準や手技の補足も提供できる。

評価者 B によるスコア				
一貫性 (Coherency)	事実性 (Factuality)	包括性 (Comprehensiveness)	安全性 (Safety)	その他コメント(適宜)
5	3	4	5	頭部 MRI での動脈瘤除外は安全性への配慮が高いと考えたが、文献に 1 つ hallucinated reference がみられた。

【症例】
42 歳、東南アジア人女性。2 週間ほど前から右眼の歪みを自覚し近医を受診、精査目的に当院紹介となった。喫煙歴なし。小児期から近視があり、現在は右-7.0 D、左-6.0 D のソフトコンタクトレンズを使用している。矯正視力は右 0.5、左 1.0、眼圧は右 14 mmHg、左 16 mmHg であった。前眼部および中間透光体に異常はない。眼底検査で両眼に豹紋状変化があり、後極部に小さな黄白色点状病変が散在している。右眼黄斑部にはわずかな出血を伴っており、一致して光干渉断層計で網膜下に辺縁不整な隆起性病変がみられた。

【質問】
あなたは眼科医で、提示されたシナリオ症例の診療情報を得たとします。
この場合、最も可能性の高い臨床診断についてその根拠を明示しながら 3 つ程度挙げて、さらに追加して行うべき検査および対応も優先度の高いものから 3 つ程度挙げてください。追加して行うべき検査および対応については、まとめて記載で構いません。
必要であれば、回答の根拠を文献からの引用で裏付けながら記載してください。文献についてはホームページのリンクを記載するか、雑誌もしくは書籍であれば代表著者、雑誌名、出版年を明記してください。
回答は常体(である調)としてまとめてください。
なお、必要に応じてウェブ検索を行っても構いません。

専門医 D の場合

1. Initial Response と評価者 E によるスコア:

近視性脈絡膜新生血管
単純出血
加齢黄斑変性

OCT
蛍光眼底造影
OCTA

滲出性変化、新生血管の有無、部位を確認する。

評価者 E によるスコア				
一貫性 (Coherency)	事実性 (Factuality)	包括性 (Comprehensiveness)	安全性 (Safety)	その他コメント(適宜)
2	2	2	2	鑑別疾患の充実に加え、検査の意義や具体的な検査内容(FA/IA など)についての言及があるとなおよいと考えた。

2. ChatGPT-4o に問いかけた Prompt:

【症例】
42 歳、東南アジア人女性。2 週間ほど前から右眼の歪みを自覚し近医を受診、精査目的に当院紹介となった。喫煙歴なし。小児期から近視があり、現在は右-7.0 D、左-6.0 D のソフトコンタクトレンズを使用している。矯正視力は右 0.5、左 1.0、眼圧は右 14 mmHg、左 16 mmHg であった。前眼部および中間透光体に異常はない。眼底検査で両眼に豹紋状変化があり、後極部に小さな黄白色点状病変が散在している。右眼黄斑部にはわずかな出血を伴っており、一致して光干渉断層計で網膜下に辺縁不整な隆起性病変がみられた。

【質問】
あなたは眼科医で、提示されたシナリオ症例の診療情報を得たとします。
この場合、最も可能性の高い臨床診断についてその根拠を明示しながら 3 つ程度挙げて、さらに追加して行うべき検査および対応も優先度の高いものから 3

つ程度挙げてください。追加して行うべき検査および対応については、まとめて記載で構いません。
必要であれば、回答の根拠を文献からの引用で裏付けながら記載してください。文献についてはホームページのリンクを記載するか、雑誌もしくは書籍であれば代表著者、雑誌名、出版年を明記してください。
回答は常体(である調)としてまとめてください。
なお、必要に応じてウェブ検索を行っても構いません。

3. Assisted Response と評価者 E によるスコア:
ポリープ状脈絡血管症（PCV）
近視性脈絡新生血管（mCNV）
中心性漿液性脈絡網膜症（CSC）

蛍光眼底造影（FA）・インドシアニングリーン蛍光造影（ICGA）
OCT アンギオグラフィー（OCT-A）
抗 VEGF 薬の検討と定期的モニタリング

東南アジア人女性で、片眼性の歪視を主訴とし、黄斑下の不整な隆起性病変および軽度の出血を伴う所見、さらに両眼に豹紋状眼底と後極部の黄白色点状病変を認めることから、ポリープ状脈絡血管症（PCV）が最も疑われる。PCV はアジア人に多く、特に女性にもみられる傾向があり、典型的に網膜下に限局性の出血や漿液性剥離を呈し、OCT で RPE 下に隆起性病変を認める。ICGA でポリープ状病変や分枝状脈絡血管網（BVN）を確認することで確定診断される。
次に考えられるのは、近視性脈絡新生血管（myopic CNV）である。高度近視歴があり（-7.0 D）、豹紋状眼底や黄白色の後極病変も両眼に存在することから近視性変化による CNV の可能性がある。ただし年齢や出血量、片眼性である点では PCV のほうが整合性が高い。
また、中心性漿液性脈絡網膜症（CSC）も鑑別に挙げられる。若年〜中年女性にもみられることがあり、OCT 上で漿液性網膜剥離として認識される。ただし、本例では出血や黄白色病変が存在しており、CSC 単独よりも他疾患の可能性が高い。
追加検査としては、まず蛍光眼底造影（FA）およびインドシアニングリーン蛍光造影（ICGA）を行い、ポリープ様病変の有無や CNV の活動性を評価する必要がある。OCT アンギオグラフィー（OCT-A）は非侵襲的に脈絡膜血管構造を可視化でき、病変の立体構造の把握に有用である。病変の活動性が確認された

場合には、抗 VEGF 薬（アフリベルセプトなど）の投与を考慮し、定期的な画像・視力モニタリングを行うことが重要である。

参考文献

Wong CW, et al. “Polypoidal choroidal vasculopathy: Evidence-based guidelines for clinical diagnosis and treatment.” Retina. 2021;41(1):1-25.
Ohno-Matsui K, et al. “Myopic choroidal neovascularization: Current management and future prospects.” Clin Ophthalmol. 2016;10:1211–1221.
Cheung CMG, et al. “Polypoidal choroidal vasculopathy: A review.” Surv Ophthalmol. 2017;62(5):786-806.
(※著者注: この 3 つの文献はいずれも著者とタイトル、発行年が一致しない hallucinated reference である)

評価者 E によるスコア				
一貫性 (Coherency)	事実性 (Factuality)	包括性 (Comprehensiveness)	安全性 (Safety)	その他コメント(適宜)
5	1	4	4	参考文献の記載があるが、いずれも fake

レジデントＦの場合

1. Initial Response と評価者Ｅによるスコア:

近視性脈絡膜新生血管、加齢黄斑変性症、

追加検査

1.FA による蛍光眼底造影

2.ICG による蛍光眼底造影

3.静的視野検査

評価者Ｅによるスコア				
一貫性 (Coherency)	事実性 (Factuality)	包括性 (Comprehensiveness)	安全性 (Safety)	その他コメント(適宜)
2	2	1	2	鑑別についての網羅性がやや低く、視野検査の必要性の言及も不十分と考えた。

2. ChatGPT-4o に問いかけた Prompt:

【症例】

42 歳、東南アジア人女性。2 週間ほど前から右眼の歪みを自覚し近医を受診、精査目的に当院紹介となった。喫煙歴なし。小児期から近視があり、現在は右-7.0 D、左-6.0 D のソフトコンタクトレンズを使用している。矯正視力は右 0.5、左 1.0、眼圧は右 14 mmHg、左 16 mmHg であった。前眼部および中間透光体に異常はない。眼底検査で両眼に豹紋状変化があり、後極部に小さな黄白色点状病変が散在している。右眼黄斑部にはわずかな出血を伴っており、一致して光干渉断層計で網膜下に辺縁不整な隆起性病変がみられた。

【質問】

あなたは眼科医で、提示されたシナリオ症例の診療情報を得たとします。

この場合、最も可能性の高い臨床診断についてその根拠を明示しながら 3 つ程度挙げて、さらに追加して行うべき検査および対応も優先度の高いものから 3 つ程度挙げてください。追加して行うべき検査および対応については、まとめて記載で構いません。

必要であれば、回答の根拠を文献からの引用で裏付けながら記載してください。文献についてはホームページのリンクを記載するか、雑誌もしくは書籍であれば代表著者、雑誌名、出版年を明記してください。

回答は常体(である調)としてまとめてください。
なお、必要に応じてウェブ検索を行っても構いません。

3. Assisted Response と評価者 E によるスコア:
a. 特発性脈絡膜新生血管（CNV）を伴う中心性漿液性脈絡網膜症（chronic CSC）または脈絡膜新生血管症（pachychoroid neovasculopathy, PNV）
● 根拠：
○ 東南アジア人、中年女性（発症好発層）
○ 両眼に豹紋状変化 → 慢性 CSC やパキコロイド関連疾患で見られる
○ OCT での網膜下の隆起性病変 → 網膜下液あるいは新生血管（type 1 CNV）を示唆
○ わずかな出血 → 新生血管の可能性
○ 歪み（変視症） → 黄斑部の病変に一致
○ 高度近視はあるが、脈絡膜萎縮の記載はなく、パキコロイドと矛盾しない

---

b. 病的近視に伴う脈絡膜新生血管（myopic CNV）
● 根拠：
○ 両眼とも強度近視（右 -7.0 D、左 -6.0 D）
○ 中年女性で発症することがある
○ 網膜下病変と出血は説明可能
○ ただし、豹紋状変化は典型的ではない点がやや矛盾

---

c. 多発後極部斑状色素上皮症（MPPEH: multifocal pattern dystrophy simulating fundus flavimaculatus）
● 根拠：
○ 両眼に小さな黄白色点状病変の散在 → パターンジストロフィーに典型的
○ 中年発症・近視歴あり
○ 視力低下、変視症の発現
○ ただし、新生血管に伴う出血が見られるのは稀

■ 優先して行うべき検査・対応（3 つ）
a. 蛍光眼底造影（FA）およびインドシアニングリーン蛍光造影（ICGA）
● CNV の有無・タイプ（classic / occult）や滲出の有無を確認
● PNV vs myopic CNV の鑑別にも有用
● ICGA ではパキベッセル（太く拡張した脈絡膜血管）を確認できる可能性あり

---

b. OCT アンギオグラフィー（OCTA）

- 非侵襲的に網膜・脈絡膜新生血管の構造的評価が可能
- type 1 CNV（網膜色素上皮下新生血管）などの評価に特に有用

c. 抗 VEGF 薬（例：アフリベルセプト、ラニビズマブなど）の導入検討

- CNV が確認された場合は視力予後改善のため速やかな治療が必要
- とくに活動性病変がある場合は早期の導入が望ましい

評価者 E によるスコア				
一貫性 (Coherency)	事実性 (Factuality)	包括性 (Comprehensiveness)	安全性 (Safety)	その他コメント(適宜)
5	2	4	5	ぶどう膜炎関連疾患の検討があるとよいと考えた。

Translations of the Representative Responses into English

【Case】

65-year-old female. She noticed double vision several months ago and visited her local ophthalmologist. She complained of double vision in both eyes, and symptoms of strabismus were suspected. She was referred to our hospital for further examination and treatment. Her medical history includes hypertension, diabetes, and herpes zoster.

Her corrected visual acuity was 1.0 in both eyes, and her intraocular pressure was 15 mmHg in the right eye and 17 mmHg in the left eye. No abnormalities were detected on slit-lamp microscopy or fundus examination. Eye position was assessed using the alternate prism cover test, showing 3 prism diopters of right/left for near vision and 6 prism diopters of esotropia plus 8 prism diopters of right/left for distance vision. A slight upward deviation of the left eye was noted during eye movement. She did not report double vision during near vision but did report it during distance vision.

【Question】

Assume you are an ophthalmologist with access to the provided patient information. Based on this scenario, please list approximately three most probable clinical diagnoses. Additionally, list approximately three recommended additional examinations and management strategies, in order of priority. If necessary, please support your answers with appropriate citations from literature (providing website links or, for journals/books, the first author, journal name, and publication year). When citing literature, please maintain a formal writing style for each response. Web searches are permitted if necessary.

In Case of Board-certified Ophthalmologist A

1. Initial Response and Scores by Grader B:

This is an elderly patient with acquired mild vertical strabismus concomitant with esotropia who strongly perceives diplopia at distance; based on the history, I suspect sagging eye syndrome. A single cranial nerve palsy does not readily explain both the left eye’s upgaze limitation and the esotropia in a unified manner. Differential diagnoses include central nervous system disorders and myasthenia gravis.

Necessary tests include brain MRI and brain CT to investigate intracranial disease and to confirm rupture of the ligament connecting the superior rectus and the lateral rectus; additional history-taking for the differential of myasthenia gravis and measurement of anti-acetylcholine receptor antibodies; and quantitative assessment of ocular motility abnormalities using the Hess red-green test. https://eyewiki.org/Sagging_Eye_Syndrome

If other diseases are excluded, management would include prescribing prism glasses and, if the patient desires, strabismus surgery to treat diplopia.

Score by Grader B				
Coherency	Factuality	Comprehensiveness	Safety	Comments
5	3	4	5	Ordering brain MRI to exclude an aneurysm reflects strong attention to safety.

2. Prompt Submitted to ChatGPT-4o:

【Case】

65-year-old female. She noticed double vision several months ago and visited her local ophthalmologist. She complained of double vision in both eyes, and symptoms of strabismus were suspected. She was referred to our hospital for further examination and treatment. Her medical history includes hypertension, diabetes, and herpes zoster.

Her corrected visual acuity was 1.0 in both eyes, and her intraocular pressure was 15 mmHg in the right eye and 17 mmHg in the left eye. No abnormalities were detected on slit-lamp microscopy or fundus examination. Eye position was assessed using the alternate prism cover test, showing 3 prism diopters of

right/left for near vision and 6 prism diopters of esotropia plus 8 prism diopters of right/left for distance vision. A slight upward deviation of the left eye was noted during eye movement. She did not report double vision during near vision but did report it during distance vision.

【Question】
Assume you are an ophthalmologist with access to the provided patient information. Based on this scenario, please list approximately three most probable clinical diagnoses. Additionally, list approximately three recommended additional examinations and management strategies, in order of priority. If necessary, please support your answers with appropriate citations from literature (providing website links or, for journals/books, the first author, journal name, and publication year). When citing literature, please maintain a formal writing style for each response. Web searches are permitted if necessary.

【Responder's draft before revision】
This is an elderly patient with acquired mild vertical strabismus concomitant with esotropia who strongly perceives diplopia at distance; based on the history, I suspect sagging eye syndrome. A single cranial nerve palsy does not readily explain both the left eye's upgaze limitation and the esotropia in a unified manner. Differential diagnoses include central nervous system disorders and myasthenia gravis.

Necessary tests include brain MRI and brain CT to investigate intracranial disease and to confirm rupture of the ligament connecting the superior rectus and the lateral rectus; additional history-taking for the differential of myasthenia gravis and measurement of anti-acetylcholine receptor antibodies; and quantitative assessment of ocular motility abnormalities using the Hess red-green test. https://eyewiki.org/Sagging_Eye_Syndrome

If other diseases are excluded, management would include prescribing prism glasses and, if the patient desires, strabismus surgery to treat diplopia.

Instruction to the model: Please revise this answer.

3. Assisted Response and Scores by Grader B:
This case raises suspicion for sagging eye syndrome (SES).

Rationale

- The patient is elderly (65 years). SES occurs more commonly in older adults due to age-related laxity of the conjunctiva and periocular supporting tissues (e.g., conjunctivochalasis and degenerative changes), and thus tends to be prevalent in this age group.
- There is a mild ocular misalignment (6Δ ET + 8Δ R/L at distance). In SES, esotropia at distance (particularly age-related distance esotropia) is typical.
- The patient complains of binocular diplopia attributable to strabismus, especially at distance, which is consistent with SES.
- Ocular motility abnormality is mild; although there is slight upgaze limitation in the left eye, the absence of marked motility restriction also fits SES.

Findings that may be inconsistent

- Slight upgaze limitation in the left eye is not a classic feature of SES. SES usually does not present with frank motility restriction; the primary issue is a malposition due to laxity of supporting tissues rather than an overt extraocular muscle palsy.
- The patient's history of diabetes and hypertension raises the possibility of microvascular cranial neuropathies, such as oculomotor nerve palsy or extraocular muscle paresis; therefore, neuromuscular etiologies other than SES should be considered.
- Although diplopia is limited to distance in this case, other causes (including cranial nerve palsies) can also manifest predominantly with distance diplopia; SES cannot be concluded solely on this basis.

Differential diagnoses

- Oculomotor nerve palsy (diabetic ischemic cranial neuropathy): Common in patients with diabetes and presents with unilateral motility limitation and diplopia; upgaze limitation raises suspicion for oculomotor involvement.
- Age-related distance esotropia (ARDE): Similar to SES; however, SES shows structural changes of orbital supporting tissues, whereas ARDE does not necessarily involve such changes.
- Myasthenia gravis: Fatigable extraocular muscle weakness causing diplopia; motility limitation and symptom severity often fluctuate diurnally.
- Thyroid eye disease: Motility restriction and diplopia due to extraocular muscle enlargement and inflammation; often accompanied by proptosis and eyelid signs.

- Brainstem disease or oculomotor nuclear lesions: May cause motility deficits and diplopia with associated neurological symptoms.

Recommended examinations

- MRI of the brain and orbits: To evaluate the state of orbital supporting structures and extraocular muscles (hypertrophy, atrophy, inflammation), to support the diagnosis of SES, and to exclude central causes.
- Detailed extraocular muscle function testing: Ocular motility assessment (including video recording) and repeated ocular alignment testing (e.g., Hess red-green test).
- Neurological evaluation: Assessment for diabetic neuropathy and oculomotor nerve palsy.
- Laboratory tests: Glycemic control, thyroid function tests (to exclude thyroid eye disease), and evaluation for myasthenia gravis (anti-acetylcholine receptor antibodies, anti-MuSK antibodies).
- Electrodiagnostic or muscle function testing (as needed): May be used to evaluate for myasthenia gravis.

Score by Grader B				
Coherency	Factuality	Comprehensiveness	Safety	Comments
5	2	5	4	The content is generally complete. It would be helpful to mention the urgency of obtaining brain MRI.

In Case of Resident C

4. Initial Response and Scores by Grader B:

a. Clinical diagnosis: Herpes zoster ophthalmicus

Rationale: Diabetes; history of herpes zoster; diplopia

b. Clinical diagnosis: Sagging eye syndrome

Rationale: Strabismus; distance 6Δ ET + 8Δ R/L; diplopia at distance

c. Clinical diagnosis: Paralytic strabismus

Rationale: Diplopia for several months; upgaze limitation

Additional tests / management

Head CT, head MRI, MRA

Serologic testing for varicella-zoster virus antibodies

Score by Grader B				
Coherency	Factuality	Comprehensiveness	Safety	Comments
2	2	2	3	It would be preferable to include mention of superior oblique palsy and oculomotor nerve palsy.

5. Prompt Submitted to ChatGPT-4o:

【Case】

65-year-old female. She noticed double vision several months ago and visited her local ophthalmologist. She complained of double vision in both eyes, and symptoms of strabismus were suspected. She was referred to our hospital for further examination and treatment. Her medical history includes hypertension, diabetes, and herpes zoster.

Her corrected visual acuity was 1.0 in both eyes, and her intraocular pressure was 15 mmHg in the right eye and 17 mmHg in the left eye. No abnormalities were detected on slit-lamp microscopy or fundus examination. Eye position was assessed using the alternate prism cover test, showing 3 prism diopters of right/left for near vision and 6 prism diopters of esotropia plus 8 prism diopters of right/left for distance vision. A slight upward deviation of the left eye was noted during eye movement. She did not report double vision during near vision but did report it during distance vision.

【Question】
Assume you are an ophthalmologist with access to the provided patient information. Based on this scenario, please list approximately three most probable clinical diagnoses. Additionally, list approximately three recommended additional examinations and management strategies, in order of priority. If necessary, please support your answers with appropriate citations from literature (providing website links or, for journals/books, the first author, journal name, and publication year). When citing literature, please maintain a formal writing style for each response. Web searches are permitted if necessary.

6. Assisted Response and Scores by Grader B:

[Top three differential diagnoses (most likely)]

a. Left superior oblique palsy (left trochlear nerve palsy, fourth nerve palsy)

This is the most likely diagnosis. The findings of diplopia more prominent at distance and reduced at near, enlargement of the strabismic angle at distance (6Δ ET + 8Δ R/L), and slight upgaze limitation in the left eye suggest decreased function of the left superior oblique muscle. Because the superior oblique primarily produces infraduction in adduction (down-and-out), its dysfunction can cause vertical and torsional diplopia. Age-related trochlear nerve palsy is relatively common, and given this patient's vascular risk factors (diabetes and hypertension), an ischemic etiology is also a differential consideration.

b. Chronic progressive external ophthalmoplegia (CPEO)

There is ocular misalignment and limitation of upgaze; the course over several months is relatively gradual, with no impact on visual acuity and without accompanying ptosis, so the presentation is not classic but remains within the differential. Note also that extraocular muscle impairment after herpes zoster has been reported (zoster ophthalmicus-related).

c. Mitochondrial disease (e.g., Kearns–Sayre syndrome)

Although age 65 is somewhat older than typical, mitochondrial syndromes that include chronic external ophthalmoplegia should be considered in the differential for progressive ocular motility limitation. Suspicion would increase if diabetes coexists or if other neurological signs appear.

[Additional examinations / management (in order of priority)]

a. Hess red-green test or Lancaster test

Useful for quantitative evaluation of ocular motility impairment and for visually identifying which muscle is affected. In superior oblique palsy, characteristic Hess

chart findings are expected (relative contraction in the affected superior oblique field with expansion of the contralateral inferior rectus field).

b. Brain MRI (particularly evaluating the brainstem and the course of the trochlear nerve)

Neuroimaging is required to exclude structural causes (brainstem infarction, tumor, aneurysm, etc.). Because the trochlear nerve exits dorsally from the brainstem and decussates, even small lesions can produce palsy. Given the vascular risk factors, assessment for ischemic lesions is also important.

c. Video recording of ocular movements; electromyography or anti-AChR antibody testing (as needed)

For differentiating other neuromuscular disorders such as myasthenia gravis. Especially useful if ocular alignment changes fluctuate diurnally or if alternating paresis of multiple muscles is suspected. While not typical in this case at present, it should be considered in the differential.

[References]

- Rosenbaum AL, Santiago AP. *Clinical Strabismus Management: Principles and Surgical Techniques*. W.B. Saunders Company, 1999.
- Walsh FB, Hoyt WF. *Clinical Neuro-ophthalmology*. Williams & Wilkins, 5th ed., 1998.
- The Japanese Society of Neuro-Ophthalmology (ed.). *Neuro-Ophthalmology*. Nankodo, 2013. (Author's note: This is a hallucinated reference; not an actual publication.)
- American Academy of Ophthalmology. *Basic and Clinical Science Course (BCSC): Neuro-Ophthalmology*. 2023–2024 edition. https://www.aao.org/

Further details on diagnostic criteria and techniques for each disease can be provided if necessary.

Score by Grader B				
Coherency	Factuality	Comprehensiveness	Safety	Comments
5	3	4	5	Ordering brain MRI to exclude an aneurysm reflects strong attention to safety; however, one hallucinated reference was noted among the citations.

【Case】

A 42-year-old Southeast Asian female. She noticed metamorphopsia in her right eye about two weeks ago and visited a local ophthalmologist and was referred to our hospital for further examination. She has no history of smoking. She has had myopia since childhood and currently wears soft contact lenses with a prescription of -7.0 D in her right eye and -6.0 D in her left eye. Her corrected visual acuity was 0.5 in her right eye and 1.0 in her left eye, and her intraocular pressure was 14 mmHg in her right eye and 16 mmHg in her left eye. No abnormalities were noted in the anterior segment or intermediate transparent media. Fundus examination revealed tigroid fundus in both eyes with small yellowish-white punctate lesions scattered in the posterior pole. The right macula showed minimal hemorrhage, and corresponding to this, optical coherence tomography revealed an irregularly shaped elevated lesion beneath the retina.

【Question】

Assume you are an ophthalmologist with access to the provided patient information. Based on this scenario, please list approximately three most probable clinical diagnoses. Additionally, list approximately three recommended additional examinations and management strategies, in order of priority. If necessary, please support your answers with appropriate citations from literature (providing website links or, for journals/books, the first author, journal name, and publication year). When citing literature, please maintain a formal writing style for each response. Web searches are permitted if necessary.

In Case of Board-certified Ophthalmologist D

1. Initial Response and Scores by Grader E:

Diagnoses

- Myopic choroidal neovascularization (mCNV)
- Simple retinal hemorrhage
- Age-related macular degeneration (AMD)

Planned tests

- OCT
- Fluorescein angiography (FA)
- OCT angiography (OCT-A)

Aim of testing

Confirm the presence, activity, and location of exudative changes and choroidal neovascularization.

Score by Grader E				
Coherency	Factuality	Comprehensiveness	Safety	Comments
2	2	2	2	In addition to expanding the differential diagnoses, it would be preferable to specify the purposes and concrete details of testing (e.g., FA/ICGA).

2. Prompt Submitted to ChatGPT-4o:

【Case】

A 42-year-old Southeast Asian female. She noticed metamorphopsia in her right eye about two weeks ago and visited a local ophthalmologist and was referred to our hospital for further examination. She has no history of smoking. She has had myopia since childhood and currently wears soft contact lenses with a prescription of -7.0 D in her right eye and -6.0 D in her left eye. Her corrected visual acuity was 0.5 in her right eye and 1.0 in her left eye, and her intraocular pressure was 14 mmHg in her right eye and 16 mmHg in her left eye. No abnormalities were noted in the anterior segment or intermediate transparent media. Fundus examination revealed tigroid fundus in both eyes with small yellowish-white punctate lesions scattered in the posterior pole. The right macula showed minimal hemorrhage, and corresponding to this, optical coherence

tomography revealed an irregularly shaped elevated lesion beneath the retina.

【Question】
Assume you are an ophthalmologist with access to the provided patient information. Based on this scenario, please list approximately three most probable clinical diagnoses. Additionally, list approximately three recommended additional examinations and management strategies, in order of priority. If necessary, please support your answers with appropriate citations from literature (providing website links or, for journals/books, the first author, journal name, and publication year). When citing literature, please maintain a formal writing style for each response. Web searches are permitted if necessary.

3. Assisted Response and Scores by Grader E:
Diagnoses
- Polypoidal choroidal vasculopathy (PCV)
- Myopic choroidal neovascularization (mCNV)
- Central serous chorioretinopathy (CSC)

Additional testing / management
- Fluorescein angiography (FA) and indocyanine green angiography (ICGA)
- OCT angiography (OCT-A)
- Consider anti-VEGF therapy and regular monitoring

Rationale
Given that this is a Southeast Asian woman presenting with unilateral metamorphopsia, an irregular, elevated submacular lesion with mild hemorrhage, and tigroid fundus with small yellow-white lesions at the posterior pole in both eyes, **PCV** is most strongly suspected. PCV is more prevalent in Asian populations, including women, and typically presents with localized subretinal hemorrhage or serous detachment, with an elevated lesion beneath the RPE on OCT. **ICGA** confirming polypoidal lesions and a branching vascular network (BVN) establishes the diagnosis.
The next consideration is **myopic CNV**. She has high myopia (−7.0 D), and the tigroid fundus and yellow-white lesions at the posterior pole in both eyes are consistent with myopic changes that can give rise to CNV. However, with respect to age, amount of hemorrhage, and unilateral presentation, PCV is more concordant.
CSC also remains in the differential. It can be seen in young to middle-aged

women and appears on OCT as serous retinal detachment. In this case, however, the presence of hemorrhage and yellow-white lesions makes an alternative diagnosis more likely than CSC alone.

Plan

As additional testing, **FA and ICGA** should first be performed to evaluate for polypoidal lesions and CNV activity. **OCT-A** can noninvasively visualize the choroidal vascular structure and is useful for understanding the three-dimensional configuration of the lesion. If lesion activity is confirmed, consider **anti-VEGF therapy** (e.g., aflibercept) and implement regular imaging and visual acuity monitoring.

References

- Wong CW, et al. “Polypoidal choroidal vasculopathy: Evidence-based guidelines for clinical diagnosis and treatment.” Retina. 2021;41(1):1-25.
- Ohno-Matsui K, et al. “Myopic choroidal neovascularization: Current management and future prospects.” Clin Ophthalmol. 2016;10:1211–1221.
- Cheung CMG, et al. “Polypoidal choroidal vasculopathy: A review.” Surv Ophthalmol. 2017;62(5):786-806.

(Author’s note: The above three citations are hallucinated in the original entry—author/title/year do not match an actual publication.)

Score by Grader B				
Coherency	Factuality	Comprehensiveness	Safety	Comments
5	1	4	4	References are listed, but all are fake.

In Case of Resident F

1. Initial Response and Scores by Grader E:

a. Clinical diagnosis: Herpes zoster ophthalmicus

Rationale: Diabetes; history of herpes zoster; diplopia

b. Clinical diagnosis: Sagging eye syndrome

Rationale: Strabismus; distance 6Δ ET + 8Δ R/L; diplopia at distance

c. Clinical diagnosis: Paralytic strabismus

Rationale: Diplopia for several months; upgaze limitation

Additional tests / management

Head CT, head MRI, MRA

Serologic testing for varicella-zoster virus antibodies

Score by Grader E				
Coherency	Factuality	Comprehensiveness	Safety	Comments
2	2	1	2	The differential diagnosis is somewhat insufficiently comprehensive, and the statement regarding the necessity of visual field testing is considered inadequate.

2. Prompt Submitted to ChatGPT-4o:

【Case】

A 42-year-old Southeast Asian female. She noticed metamorphopsia in her right eye about two weeks ago and visited a local ophthalmologist and was referred to our hospital for further examination. She has no history of smoking. She has had myopia since childhood and currently wears soft contact lenses with a prescription of -7.0 D in her right eye and -6.0 D in her left eye. Her corrected visual acuity was 0.5 in her right eye and 1.0 in her left eye, and her intraocular pressure was 14 mmHg in her right eye and 16 mmHg in her left eye. No abnormalities were noted in the anterior segment or intermediate transparent media. Fundus examination revealed tigroid fundus in both eyes with small yellowish-white punctate lesions scattered in the posterior pole. The right macula

showed minimal hemorrhage, and corresponding to this, optical coherence tomography revealed an irregularly shaped elevated lesion beneath the retina.

【Question】
Assume you are an ophthalmologist with access to the provided patient information. Based on this scenario, please list approximately three most probable clinical diagnoses. Additionally, list approximately three recommended additional examinations and management strategies, in order of priority. If necessary, please support your answers with appropriate citations from literature (providing website links or, for journals/books, the first author, journal name, and publication year). When citing literature, please maintain a formal writing style for each response. Web searches are permitted if necessary.

3. Assisted Response and Scores by Grader E:

a. Chronic central serous chorioretinopathy (CSC) with secondary/idiopathic choroidal neovascularization (CNV), or pachychoroid neovasculopathy (PNV)

Rationale:

- Middle-aged Southeast Asian woman (within a commonly affected demographic)
- Tigroid changes in both eyes → seen in chronic CSC/pachychoroid-spectrum disease
- Elevated subretinal lesion on OCT → suggests subretinal fluid and/or neovascularization (type 1 CNV)
- Mild hemorrhage → supports the possibility of neovascularization
- Metamorphopsia → consistent with a macular lesion
- Although high myopia is present, choroidal atrophy is not described; this does not contradict a pachychoroid process

b. Myopic choroidal neovascularization (myopic CNV)

Rationale:

- High myopia in both eyes (−7.0 D OD, −6.0 D OS)
- Can present in middle-aged women
- Subretinal lesion and hemorrhage can be explained by mCNV
- However, the tigroid changes are not entirely typical, which is somewhat contradictory

c. Multifocal pattern dystrophy simulating fundus flavimaculatus (MPDSFF;

“MPPEH”)

Rationale:

- Scattered small yellow-white lesions in both posterior poles → typical of pattern dystrophy
- Onset in middle age with a history of myopia
- Visual decrease and metamorphopsia
- However, hemorrhage due to CNV is uncommon in this entity

Priority additional examinations / management (three items)

a. Fluorescein angiography (FA) and indocyanine green angiography (ICGA)

- Confirm presence/type of CNV (classic/occult) and exudation
- Useful in differentiating PNV vs. myopic CNV
- ICGA may demonstrate pachyvessels (dilated, thickened choroidal vessels)

b. OCT angiography (OCTA)

- Noninvasive structural assessment of retinal/choroidal neovascularization
- Particularly useful for evaluating type 1 CNV (sub-RPE neovascularization)

c. Consider initiation of anti-VEGF therapy (e.g., aflibercept, ranibizumab)

- If CNV is confirmed, prompt treatment is needed to improve visual prognosis
- Early initiation is desirable when the lesion is active

Score by Grader E				
Coherency	Factuality	Comprehensiveness	Safety	Comments
5	2	4	5	It would be helpful to consider uveitis-related diseases as part of the differential.
