# Supplementary material for: Evolving Consultation: Enhancing Ophthalmic Diagnostic Performance Using Large Language Model
Source: Ophthalmol Sci. 2025 Nov 11;6(2):101004. doi: 10.1016/j.xops.2025.101004 (PMC12919258; doi:10.1016/j.xops.2025.101004)
Supplement: Table S2 [file mmc4.pdf]

Table S2. Inter-Rater Reliability among the Three Graders for Each Evaluation Metric.

Metrics	ICC(2,1)	95 % CI [lower, upper]
Coherency	0.96	[0.95, 0.97]
Factuality	0.98	[0.98, 0.98]
Comprehensiveness	0.89	[0.88, 0.91]
Safety	0.89	[0.87, 0.91]

ICC = intraclass correlation coefficient; CI = confidence interval.
