# Supplementary material for: Evolving Consultation: Enhancing Ophthalmic Diagnostic Performance Using Large Language Model
Source: Ophthalmol Sci. 2025 Nov 11;6(2):101004. doi: 10.1016/j.xops.2025.101004 (PMC12919258; doi:10.1016/j.xops.2025.101004)
Supplement: Table S3 [file mmc5.pdf]

Table S3. Literature Citations and Hallucinated References Before and After ChatGPT-4o Assistance

Group	Response	Total citations	Accurate references (%)	Hallucinated references (%)	*P*-value
Resident	Initial	24	24 (100%)	0 (0%)	—
	Assisted	98	55 (56%)	43 (44%)	<0.001*
Board-Certified	Initial	12	12 (100%)	0 (0%)	—
	Assisted	68	38 (56%)	30 (44%)	0.003*
ChatGPT-4o (reference)	—	84	80 (95%)	4 (5%)	—

P-values indicate comparisons of hallucinated reference proportions between initial and assisted responses within each ophthalmologist group, calculated by Fisher's exact test.
The ChatGPT-4o-generated response data are provided for comparative reference only and were not statistically tested against physician responses.
*$P < 0.05$.
